# Acceleration of pCASL-Based Cerebral 4D MR Angiography Using Compressed SENSE: A Comparison With SENSE

**DOI:** 10.3389/fneur.2022.796271

**Published:** 2022-03-21

**Authors:** Maoxue Wang, Yiming Ma, Fei Chen, Fei Zhou, Jilei Zhang, Bing Zhang

**Affiliations:** ^1^Department of Radiology, The Affiliated Drum Tower Hospital of Nanjing University Medical School, Nanjing, China; ^2^Department of Radiology, The Yancheng School of Clinical Medicine of Nanjing Medical University, Yancheng, China; ^3^Philips Healthcare, Shanghai, China; ^4^Institute of Brain Science, Nanjing University, Nanjing, China

**Keywords:** Compressed SENSE, SENSE, magnetic resonance angiography, cerebrovascular, arterial spin labeling

## Abstract

**Objectives:**

The objectives of this study were to accelerate the non-contrast-enhanced four-dimensional magnetic resonance angiography (4D MRA) based on pseudocontinuous arterial spin labeling combined with the Keyhole and View-sharing (4D-PACK) procedure using the Compressed SENSE (C-SENSE) and to improve intracranial vasculopathy evaluations for clinical purposes.

**Methods:**

4D-PACK acquisition with different C-SENSE and SENSE acceleration factors was performed on 29 healthy volunteers and six patients by means of a 3.0 T MR system. Two radiologists used a 4-grade scale to qualitatively assess the vessel visualization of the middle cerebral artery (MCA) and used a 5-grade scale to qualitatively examine the image quality of 4D-PACK axial source images. Interobserver agreement was assessed by determining the weighted kappa statistic. The contrast-to-noise ratio (CNR) and arterial transmit time (ATT) were calculated in four segments of the MCA. The repeated measures one-way ANOVA for CNR and the Friedman test for source images and vessel visualization were used to analyse the differences in five sequences.

**Results:**

(1) At the M4 segment, C-SENSE5 acquisition (scores, 2.72 ± 0.53) and C-SENSE6.5 (scores, 2.55 ± 0.57) provided similar vessel visualization compared with SENSE4.5 (scores, 2.72 ± 0.46); however, C-SENSE8 (scores, 1.79 ± 0.49) and C-SENSE10 (scores, 1.52 ± 0.51) had lower scores (*P* < 0.050). (2) The source image quality of C-SENSE5 (scores, 4.55 ± 0.51), C-SENSE6.5 (scores, 4.03 ± 0.33), and C-SENSE8 (scores, 3.48 ± 0.51) acquisition was higher than that of SENSE4.5 (scores, 3.07 ± 0.26) (*P* < 0.001). (3) CNRs of different MCA segments for C-SENSE5 and C-SENSE6.5 acquisitions were not significantly different compared with that of SENSE4.5 acquisition. However, the CNRs were significantly lower for C-SENSE8 (M1: 45.85 ± 13.91, M2: 27.08 ± 9.92, M4: 7.93 ± 4.49) and C-SENSE10 (M1: 37.94 ± 9.92, M2: 23.51 ± 9.0, M4: 6.78 ± 4.12) than for SENSE4.5 (M1: 55.49 ± 13.39, M2: 36.94 ± 11.02, M4: 10.18 ± 5.15) in each corresponding segment (*P* < 0.050). ATTs in all MCA segments within different accelerating C-SENSE factors were obviously correlated with SENSE4.5.

**Conclusion:**

C-SENSE6.5 acquisition could be used to evaluate both the intracranial macrovascular and distal arteries, which could reduce the acquisition time by 18% (5 min 5 s) compared with SENSE4.5. Moreover, C-SENSE8 acquisition (37% acceleration, 3 min 54 s) could be used for routine screening and clinical diagnosis of intracranial macrovascular disease with balanced image quality.

## Introduction

Four-dimensional magnetic resonance angiography (4D MRA) provides dynamic flow information compared with three-dimensional (3D) MRA and is crucial in the evaluation of intracranial vasculopathy outcomes, such as arteriovenous malformation (AVM), arteriovenous fistula (AVF), and intracranial aneurysm (1–4). Non-contrast-enhanced (NCE) 4D MRA can be acquired based on the arterial spin labeling (ASL) technique, which has been used for monitoring disease progression or in postoperative follow-up evaluation of intracranial vasculopathy due to its high spatial and temporal resolution (5–7). However, the ASL-based 4D MRA cannot be widely used in clinical applications because high-quality images at multiple time points are difficult to obtain within an acceptable acquisition time.

Recently, Compressed SENSE (C-SENSE) was developed as a start-of-the-art reconstruction algorithm that allows the combination of wavelet transformation of compressed sensing (CS) with the coil information of SENSE; additionally, this algorithm has been used to accelerate MRI techniques, especially 3D acquisition (8–10). Highly accelerated vessel-selective arterial spin labeling angiography was sampled in two dimensions (2D) using a golden angle radial trajectory and reconstructed by parallel imaging and a compressed sensing framework (11). Zhou et al. proposed an accelerated NCE 4D MRA technique that does not compromise the image quality and the temporal fidelity using golden-angle stack-of-stars trajectory and CS with magnitude subtraction (12). However, this technique uses non-Cartesian sampling and is not widely available for clinical use. Other undersampled acquisition techniques, including CENTRA with the keyhole method, partial Fourier transform, and SENSE, have been used to shorten the scan time of 4D MRA acquisition (13). Recently, a fast 4D MRA sequence based on pseudocontinuous ASL (pCASL) combined with CENTRA k-space sampling with the keyhole and view-sharing techniques (4D-PACK) was developed to evaluate intracranial vasculopathy (14). Previous findings suggested that the 4D-PACK images have a higher contrast-to-noise ratio (CNR) and offer better visualization of the distal cerebral arteries than images acquired by contrast inherent inflow-enhanced multiphase angiography (CINEMA) in patients with moyamoya disease (MMD) (5). To date, the combination of C-SENSE and the Keyhole technique has not been examined as a way to accelerate 4D MRA acquisition.

In this study, we propose a protocol to further accelerate 4D MRA using 4D-PACK combined with different C-SENSE factors and evaluate the image quality compared with SENSE acceleration by qualitative and quantitative analyses.

## Methods and Materials

### 4D-PACK With Compressed SENSE

The 4D-PACK technique using pCASL combined with Keyhole and View-sharing for acceleration is similar to techniques used in previous work (14). Image acquisition at each time point included presaturation, control or labeled modules, and post labeling delay, followed by data acquisition. Inflow dynamic imaging was performed by increasing the label duration in this study, as described in previous studies (14, 15), rather than by changing the post labeling delay. The label duration type is considered to be advantageous in peripheral artery visualization (5). Thus, the CENTRA keyhole and view-sharing techniques were used to accelerate 4D pCASL acquisition for clinical use (14).

To further accelerate the acquisition time of 4D-PACK, the separated k-space filling based on the CENTRA keyhole technique of each time point of 4D-PACK could be combined with the C-SENSE technique. The C-SENSE technique was a combination of SENSE and CS by leveraging a balanced variable density incoherent undersampling acquisition scheme and iterative reconstruction algorithm (16, 17). The reconstruction algorithm of the C-SENSE technique is as follows (18):


(1) $$\begin{array}{l} p=\min(\sum_{i=1}^{\#coils}∥m_{d,i}-ES_{d,i}p{∥}_{2}^{2}+\lambda_{1}∥R^{-1\backslash 2}p{∥}_{2}^{2}\;\\ \;+\lambda_{2}∥\psi_{p}{∥}_{1}) \end{array}$$

where *p* is the reconstructed image, *m*_*d,i*_ and *S*_*d,i*_ are the measured value and the coil sensitivity for a given coil element after noise decorrelation, respectively, E is the undersampling Fourier operator defined by the subsampling pattern, λ_1_ is the regularization factor for balancing between prior knowledge of the image content and data consistency, R is the coarse resolution data acquired with the SENSE reference scan from the integrated body coil, which is used to constrain the solution during the regularization process, λ_2_ is the regularization factor used to balance data consistency and the sparsity constraint in the iterative construction, and ψ is the sparsity transform into the wavelet domain.

### MR Experiments

The institutional review board approved this study (2021-026-02), and informed consent was obtained from all participants. Twenty-nine healthy volunteers (11 men, 43.17 ± 14.83 years old) and a patient with AVM and five patients with MMD from one institution were enrolled in this prospective study. The exclusion criteria of healthy volunteers were any stenosis or occlusion of the intracranial artery, pregnancy, contradiction to MRI, or any metal or motion artifact of MRI scans.

All participants were evaluated with 4D-PACK with five acceleration factors on an Ingenia CX 3.0T system (Philips Healthcare, Best, The Netherlands) using a 32-element phased-array head coil. The positions of all sequences with whole-brain coverage were the same in every subject. The parameters of 4D-PACK were as follows: 3D T1 turbo field echo (TFE) acquisition; repetition time/echo time (TR/TE) = 6/1.93 ms; field of view (FOV) = 210 × 210 × 120 mm^2^; flip angle = 11°; resolution = 1 × 1.4 × 1.6 mm^2^; keyhole percentage: 75%; 8 time points with different label durations = 100, 200, 400, 600, 800, 1,200, 1,600, 2,200 ms; and post labeling delay = 50 ms. The labeling module of pCASL utilizes a train of discrete RF pulses to mimic the flow-driven adiabatic inversion. The RF pulses are played in the presence of a switching slice-select gradient, which defines the labeling plane, and the parameters are as follows: flip angle = 22°, B_1_ = 6 μT, pulse duration=0.48 ms, gradient strength= 5 mT/m, and slew rate = 13.7 mT/m/ms. Spins moving perpendicular through the labeling plane experience a flow-driven adiabatic inversion. 4D-PACK with a SENSE reduction was used as the reference because previous studies have demonstrated the clinical value of peripheral visualization in healthy controls, patients with AVM, and patients with MMD (5, 14, 19). 4D-PACK sequences with four different C-SENSE factors (5, 6.5, 8, and 10 corresponding to scan time of 6 min 13 s, 5 min 15 s, 3 min 54 s, and 3 min 5 s) were compared with that of SENSE4.5 (P reduction = 3 and S reduction = 1.5, scan time = 6 min 13 s). Axial maximum intensity projection (MIP) images at each time point were generated with a full volume thickness of 120 mm for 4D-PACK with different acceleration factors. All images were transmitted to the Philips MR workstation (IntelliSpace Portal, ISP) for analysis.

### Data Analysis

#### Qualitative Analysis of MIP and Source Images of 4D-PACK

The image quality of 4D-PACK scans was assessed by two radiologists (WM and CF, with 10 and 9 years of experience, respectively) independently. Any disagreement was resolved by discussion to reach consensus.

Visualization of the middle cerebral artery (MCA) at M1, M2, M3, and M4 on MIP images was evaluated on a 4-point scale: grade 1, vessel disappearance; grade 2, heavily blurred vessel margins with no confident diagnosis; grade 3, slightly vague vessel margin but a confident diagnosis; grade 4, sharp vessel margins with a confident diagnosis (20). The M1 segment originates from the terminal bifurcation of the internal carotid artery and terminates at the main bifurcation. The M2 segment was defined as the arteries from the main bifurcation to the circular sulcus of the insula. The M3 segment of the MCA was defined as the arteries from the insula to the cortex. The M4 segment encompasses the cortical arteries originating from the Sylvian fissure and going to the cortex of the brain (21).

The quality of axial source images (label and control images) was assessed on a 5-point scale: grade 1, non-diagnostic image with severe artifacts and image distortion; grade 2, poor diagnostic image with obvious artifacts and image distortion; grade 3, moderate image artifacts and image distortion; grade 4, good diagnostic image with few artifacts and mild image distortion; grade 5, excellent image quality with almost no artifacts or image distortion (22).

#### Quantitative Analysis of CNR and Arterial Transmit Time (ATT) Measurements

The CNR of the MCA was measured in five sequences by the same neuroradiologist with 10 years of experience (WM). Circular or oval regions of interest (ROIs; 3–10 mm^2^) were located on the M1, M2, M3, and M4 segments of the MCA and background tissues in the white matter on axial MIP images. ROIs were drawn at the first time points and copied to others for a single 4D-PACK sequence. Almost the same position in different sequences was ensured manually. There were two ROIs in each of the M1 and M4 segments and one in each of the M2 and M3 segments at each timepoint. The CNR was calculated as follows: CNR = (S_MCA_-S_WM_)/SD_WM_, where S_MCA_ is the average signal of ROIs at each time point in one sequence, and S_WM_ and SD_WM_ are the average signal and SD of background tissues at each time point, respectively.

The arterial transmit times **(**ATTs) for the MCA in the five groups were calculated to validate the dynamic inflow data. The 6 ROIs were located on the MCA at each time point. The maximum signal within each time point was measured, and the ATT was defined as the time required for the signal to reach its half maximum value, which was determined according to a previous report (14).

### Patient Evaluation

SENSE4.5, C-SENSE5, C-SENSE6.5, C-SENSE8, and C-SENSE10 acquisition was performed on a patient (female, 50 years old) with AVM and five patients (four females, aged 30–55 years) with MMD. The 4D-PACK images were compared with digital subtraction angiography (DSA) images.

### Statistical Analysis

An interobserver agreement for vessel visualization and source image quality was determined by the weighted kappa statistic. The agreement was interpreted based on the kappa value as follows: ≤0.20, slight; 0.21–0.40, fair; 0.41–0.60, moderate; 0.61–0.80, substantial; and 0.81–1.00, almost perfect. Repeated measures one-way ANOVA and the Friedman test were used to evaluate the differences in CNRs, vessel visualization, and source image quality of 4D-PACK images based on five different acceleration factors, and *post-hoc* Bonferroni tests were performed. The Bland–Altman analysis was performed to assess the associations of ATTs in five sequences. A statistical analysis was performed with the SPSS 25.0 software (IBM). *P* < 0.050 was considered statistically significant.

## Results

### Qualitative Results of MIP and Source Images

**Figure 1:**
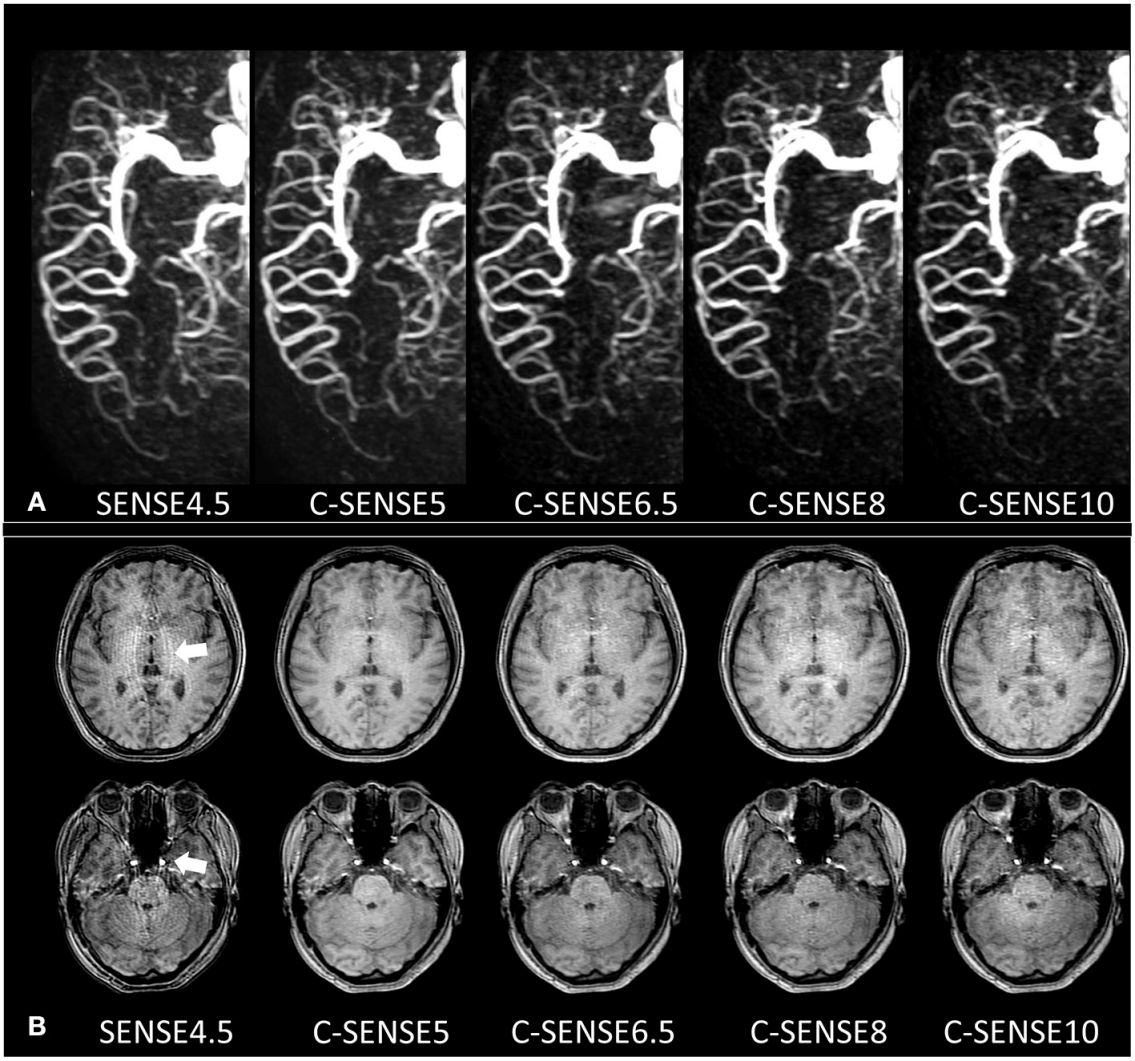
**(A)** 4D-PACK images with different acceleration factors in a 32-year-old woman. The M1, M2, and M3 segments of the MCA had 4 scores in all sequences and had sharp vessel margins with confident diagnosis. The M4 segment in SENSE4.5 and C-SENSE5 had 3 scores, which indicated a slightly vague vessel margin but resulted in a confident diagnosis. The M4 segment in C-SENSE6.5 and C-SENSE8 had 2 scores, which indicated heavily blurred vessel margins with no confident diagnosis. The M4 segment in C-SENSE10 had a score of 1 because some vessels disappeared. **(B)** Source images of 4D-PACK combined with different acceleration factors. SENSE4.5 and C-SENSE10 acquisitions (3 scores) had moderate artifacts; there were almost no artifacts in C-SENSE5 acquisition (5 scores), and there were a few artifacts when C-SENSE6.5 and C-SENSE8 (4 scores) were used.

The interobserver agreement of vessel visualization before consensus was substantial for the M3 (kappa = 0.793) and M4 (kappa = 0.775) segments and almost perfect for the M1 (kappa = 0.845) and M2 (kappa = 0.813) segments of the MCA. Vessel visualization in the M1 (scores, 3.88 ± 0.32), M2 (scores, 3.97± 0.18), and M3 (scores, 3.97 ± 0.16) segments was better than that of the M4 (scores, 2.27± 0.71) segment. Vessel visualization in the M1, M2, and M3 segments showed no significant differences among sequences. However, for vessel visualization at the M4 segment, C-SENSE5 (scores, 2.72 ± 0.53) and C-SENSE6.5 (scores, 2.55 ± 0.57) were similar to SENSE4.5 (scores, 2.72 ± 0.46) and better than C-SENSE8 (scores, 1.79 ± 0.49) and C-SENSE10 (scores, 1.52 ± 0.51) (*P* < 0.001) (Figure 1, Tables 1, 2).

**Table 1:** Results of source image, vessel visualization, and contrast-to-noise ratio of M1, M2, M3, and M4 segments of the middle cerebral artery when evaluated with 4D-PACK accelerated with SENSE4.5, C-SENSE5, C-SENSE6.5, C-SENSE8, and C-SENSE10 (mean value ± SD).

	**SENSE4.5**	**C-SENSE5**	**C-SENSE6.5**	**C-SENSE8**	**C-SENSE10**	***p***
Source image	3.07 ± 0.26	4.55 ± 0.51	4.03 ± 0.33	3.48 ± 0.51	3.07 ± 0.26	<0.001
Vessel visualization						
M1	3.83 ± 0.38	4 ± 0	3.90 ± 0.31	3.79 ± 0.41	3.90 ± 0.31	0.145
M2	4 ± 0	3.93 ± 0.26	3.90 ± 0.31	4 ± 0	4 ± 0	0.092
M3	4 ± 0	3.97 ± 0.19	3.90 ± 0.31	4 ± 0	4 ± 0	0.075
M4	2.72 ± 0.46	2.72 ± 0.53	2.55 ± 0.57	1.79 ± 0.49	1.52 ± 0.51	<0.001
CNR						
M1	55.49 ± 13.39	55.39 ± 19.41	52.61 ± 17.28	45.85 ± 13.91	37.94 ± 9.92	<0.001
M2	36.94 ± 11.02	35.34 ± 12.20	32.95 ± 11.34	27.08 ± 9.92	23.51 ± 9.0	<0.001
M3	21.06 ± 8.95	18.24 ± 10.16	19.12 ± 10.08	17.30 ± 10.43	13.63 ± 6.59	0.002
M4	10.18 ± 5.15	10.90 ± 6.0	9.74 ± 5.10	7.93 ± 4.49	6.78 ± 4.12	<0.001

*The p-value is the result of repeated measures one-way ANOVA for contrast-to-noise, Friedman test for source image and vessel visualization*.

**Table 2:** Exact *p*-value of *post-hoc* analysis in significantly different Friedman test and repeated measures one-way ANOVA.

	**4.5 vs. 5**	**4.5 vs. 6.5**	**4.5 vs. 8**	**4.5 vs. 10**	**5 vs. 6.5**	**5 vs. 8**	**5 vs. 10**	**6.5 vs. 8**	**6.5 vs. 10**	**8 vs. 10**
Source image	<0.001	<0.001	0.042	0.967	0.062	<0.001	<0.001	0.003	<0.001	0.038
Vessel visualization										
M4	0.934	0.361	<0.001	<0.001	0.406	<0.001	<0.001	<0.001	<0.001	0.299
CNR										
M1	1	1	<0.001	<0.001	1	0.002	<0.001	0.006	<0.001	<0.001
M2	1	0.624	<0.001	<0.001	1	0.001	<0.001	0.021	<0.001	0.06
M3	1	1	0.455	0.001	1	1	0.093	1	0.052	0.279
M4	1	1	0.003	<0.001	0.47	<0.001	<0.001	0.009	<0.001	0.05

*4.5: SENSE4.5; 5: C-SENSE5; 6.5: C-SENSE6.5; 8: C-SENSE8; 10: C-SENSE10*.

**Figure 2:**
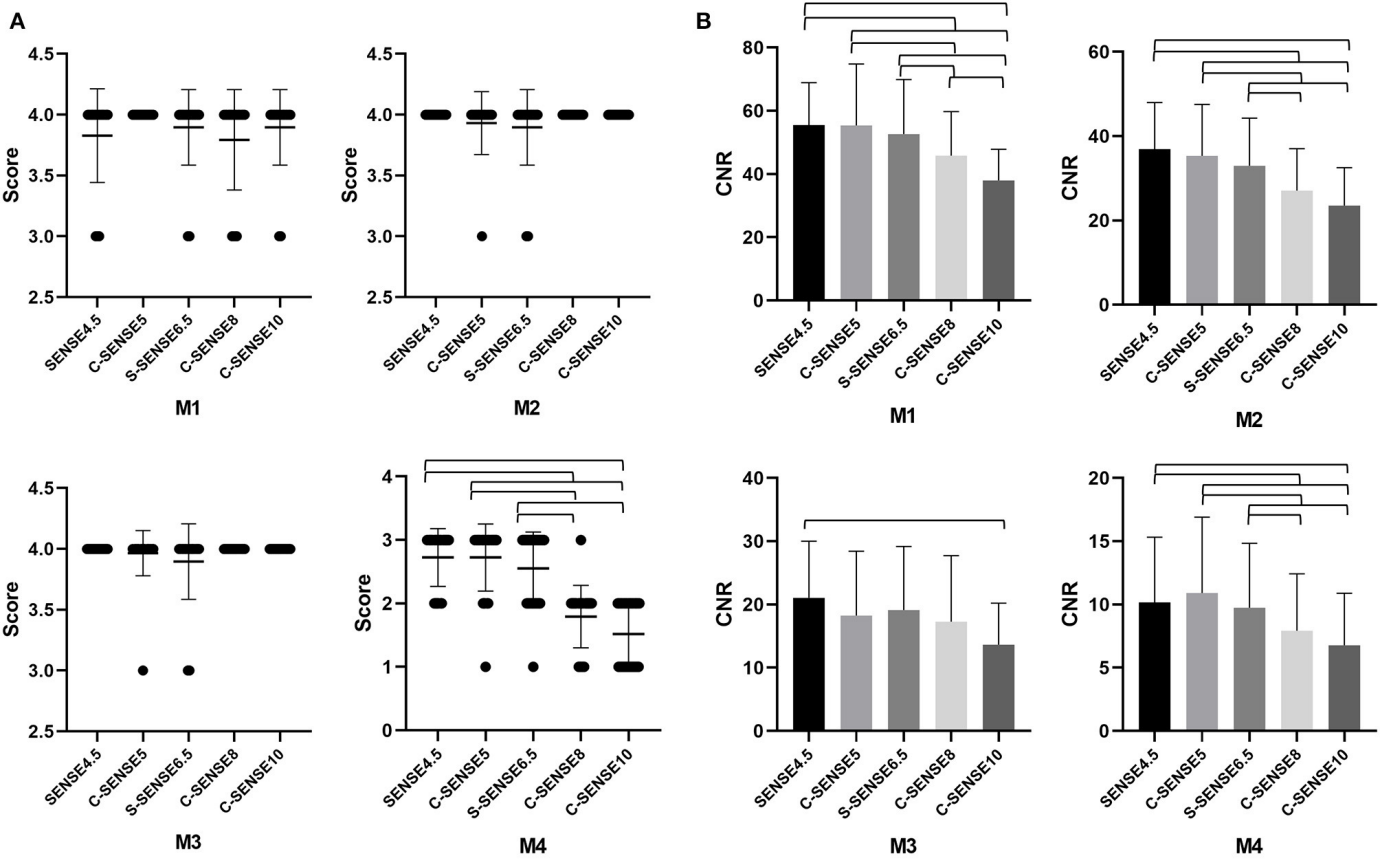
**(A,B)** The mean vessel visualization scores and CNR of M1, M2, M3, and M4 segments of the MCA in images acquired by 4D-PACK with different accelerated factors. The error bars are the standard deviation of the regions of interest (ROIs). The parentheses indicate that vessel visualization scores or CNR in the two sequences are significantly different (*P* < 0.05).

The interobserver agreement of source image quality before consensus was substantial for SENSE4.5 (kappa = 0.633) and C-SENSE10 (kappa = 0.651) acquisition and almost perfect for C-SENSE6.5 (kappa = 0.862), C-SENSE5 (kappa = 0.843), and C-SENSE8 (kappa = 0.863) acquisition. The source image quality scores of C-SENSE5 (scores, 4.55 ± 0.51), C-SENSE6.5 (scores, 4.03 ± 0.33), and C-SENSE8 (scores, 3.48 ± 0.51) acquisitions were higher than those obtained with SENSE4.5 (scores, 3.07 ± 0.26) and C-SENSE10 (scores, 3.07 ± 0.26) (*P* < 0.001) (Figures 1, 2, Tables 1, 2).

### Quantitative CNR and ATT Data

The CNRs of all the tested sequences are shown in Table 1 and Figure 2. The results of the *post-hoc* analysis are listed in Table 2. The CNRs of different MCA segments for C-SENSE5 and C-SENSE6.5 acquisitions were not significantly different compared with that of SENSE4.5 acquisition. However, the CNRs were significantly lower for C-SENSE8 (M1: 45.85 ± 13.91, *P* < 0.001; M2: 27.08 ± 9.92, *P* < 0.001; M4: 7.93 ± 4.49, *P* = 0.003) and C-SENSE10 (M1: 37.94 ± 9.92, *P* < 0.001; M2: 23.51 ± 9.0, *P* < 0.001; M4: 6.78 ± 4.12, *P* < 0.001) than for SENSE4.5 (M1: 55.49 ± 13.39, M2: 36.94 ± 11.02, M4: 10.18 ± 5.15) in each corresponding segment. The CNR of the M3 segment in the C-SENSE10 (13.63 ± 6.59) acquisition was also lower than that in the SENSE4.5 acquisition (21.06 ± 8.95, *P* = 0.001).

**Figure 3:**
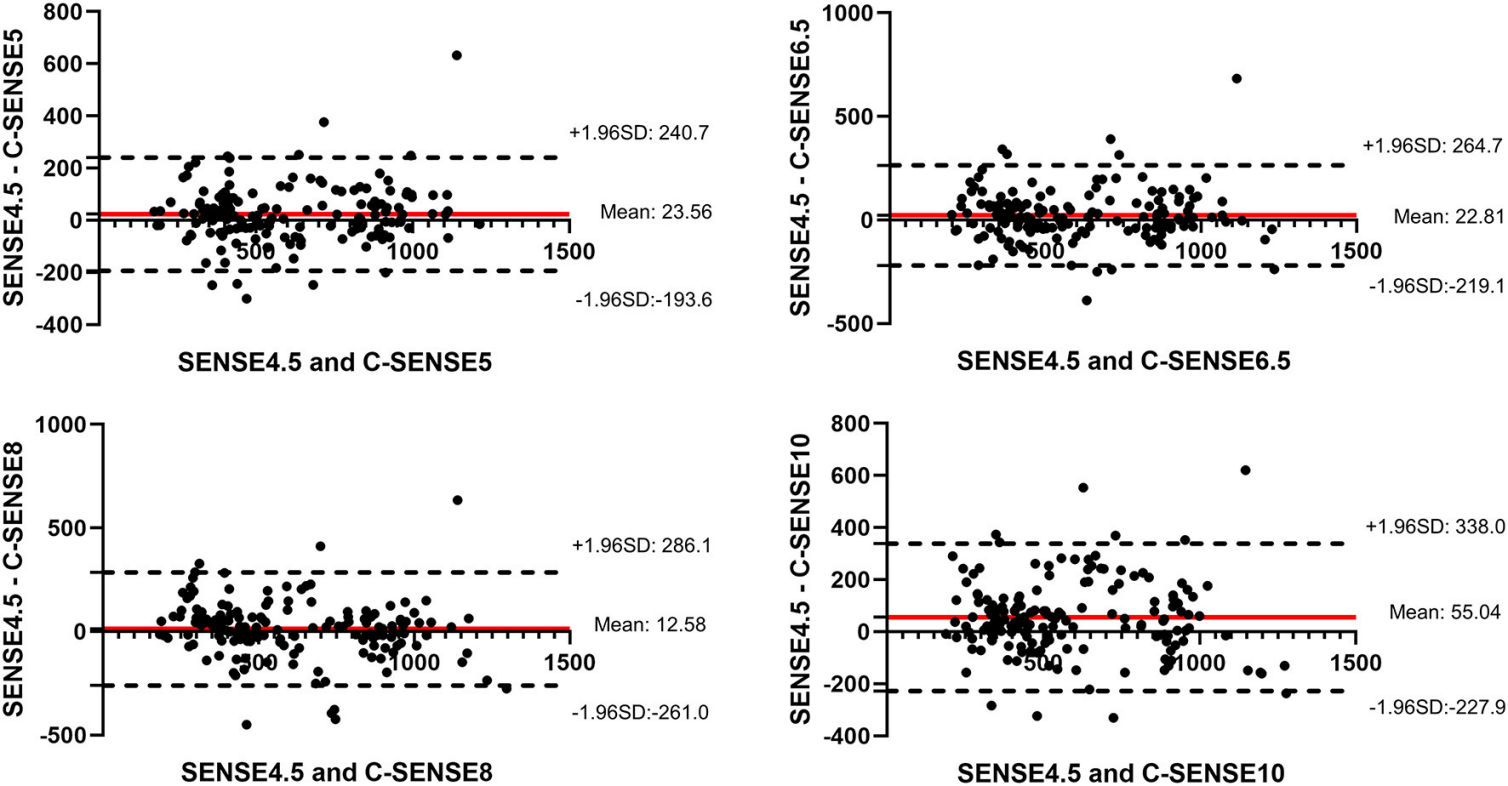
Bland–Altman plots together with 95% CI of arterial transmit time measurements from all ROIs in all volunteers.

Bland–Altman plots together with 95% CI of ATT from all subjects between each C-SENSE sequence and SENSE4.5 in the M1, M2, M3, and M4 segments are shown in Figure 3. Regarding each C-SENSE sequence and SENSE4.5, retests for ATT displayed good correlation for most ROIs.

### Patient Validation

**Figure 4:**
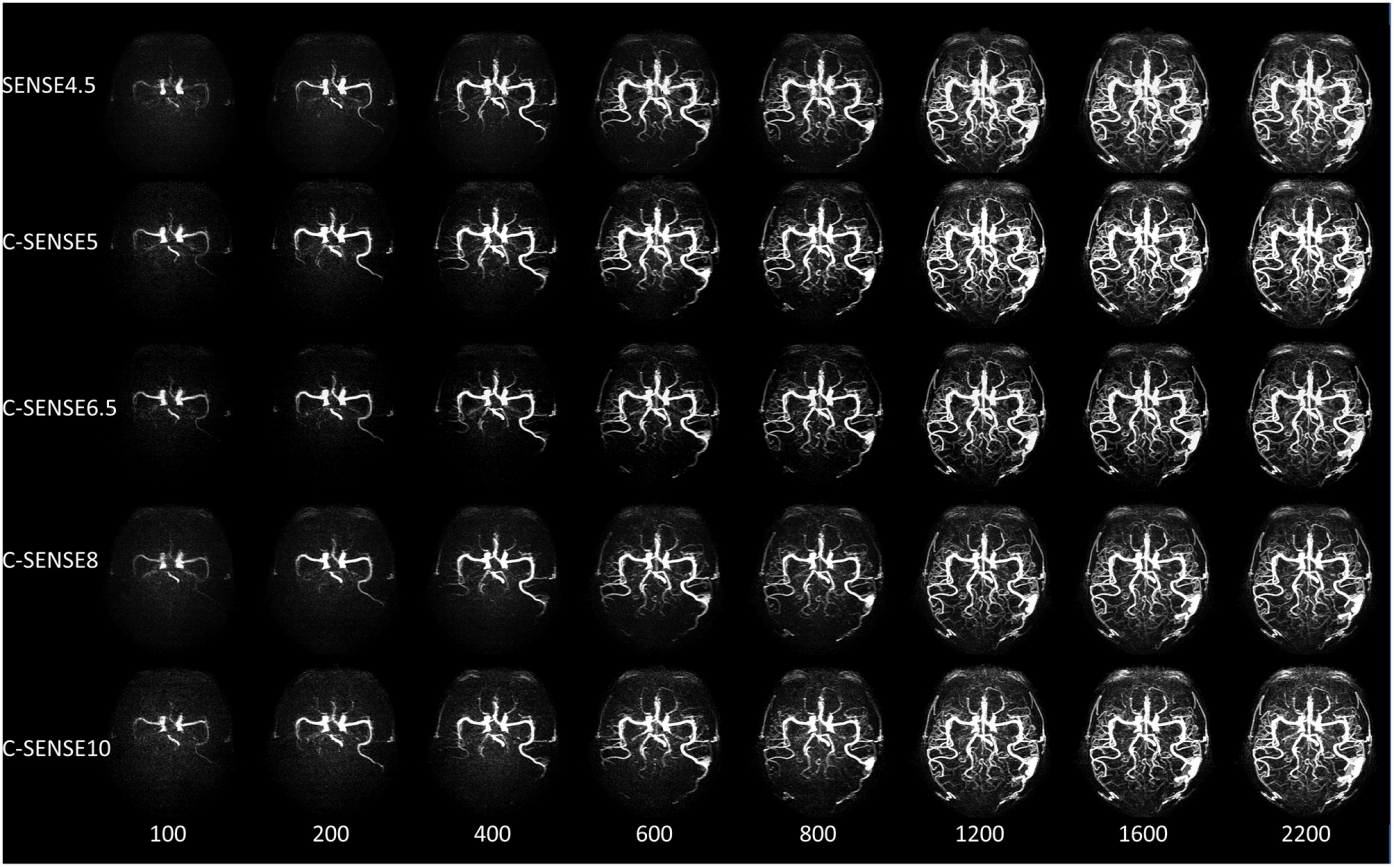
A 50-year-old woman with arterial venous malformation. The feeding artery, the nidus, and the draining vein were clearly visible from 600 to 2,200 ms in all sequences, while the distal vessels of the middle cerebral artery had vague margins in images acquired by C-SENSE8 and C-SENSE10 vs. SENSE4.5, C-SENSE5, and C-SENSE6.5 acquisitions.

Figure 4 shows the 4D-PACK images combined with different acceleration factors in a patient with cerebral AVM. The feeding artery was a branch of the MCA. The nidus appeared at the fourth stage, and the draining vein appeared at the sixth stage. The feeding artery, the nidus, and the draining vein were clearly visible on 4D-PACK images with all five acceleration factors. The feeding artery, nidus, and draining vein were confirmed by DSA.

**Figure 5:**
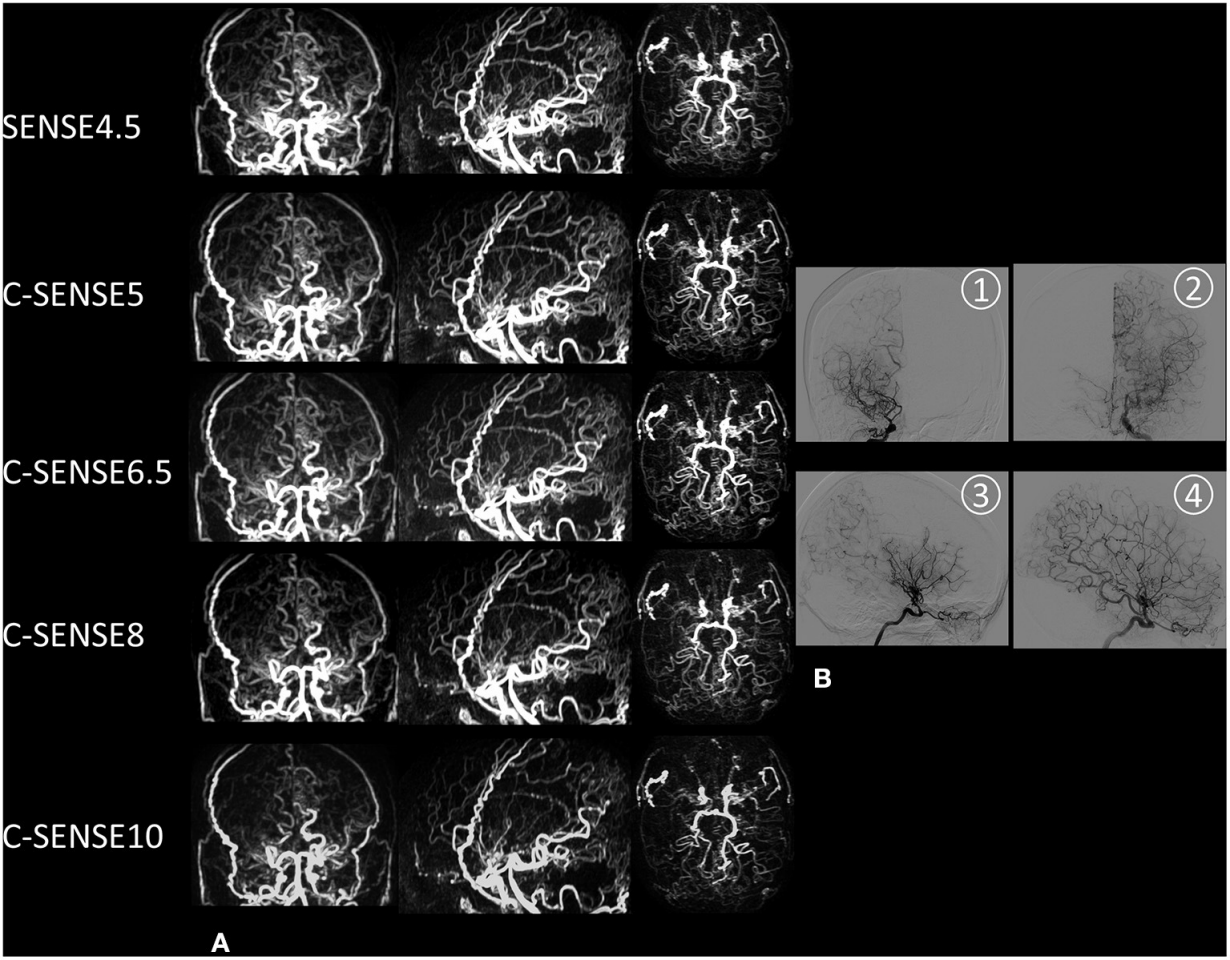
A 50-year-old woman with moyamoya disease. **(A)** Coronal, sagittal, and axial maximum intensity projection images acquired by 4D-PACK combined with different acceleration factors. SENSE4.5, C-SENSE5, and C-SENSE6.5 acquisitions provided better visualization of distal arteries and collaterals than C-SENSE8 and C-SENSE10. The distal artery is slightly blurred in images acquired by C-SENSE8, and some of them disappear in images acquired by C-SENSE10. **(B)** Anteroposterior and lateral digital subtraction angiography images of the right (1, anteroposterior view; 3, lateral view) and left (2, anteroposterior view; 4, lateral view) internal carotid arteries. The bilateral middle cerebral arteries are occluded and surround many collaterals.

Figure 5 shows 4D-PACK images with all five acceleration factors and DSA images of the bilateral internal carotid artery in a patient with MMD. Vessel visualization of the distal artery and collaterals was almost the same for the use of C-SENSE5 and C-SENSE6.5 acquisition compared with the use of SENSE4.5. Some terminal arteries had vague margins when visualized by C-SENSE8. Few terminal vessels were shown by C-SENSE10 compared with DSA images. The results indicate that C-SENSE5 and C-SENSE6.5 acquisition provided vessel visualization similar to that provided by SENSE4.5.

## Discussion

To our knowledge, this is the first study to investigate the feasibility of ASL-based 4D MRA acceleration with a combination of 4D-PACK and C-SENSE. In this study, C-SENSE5 and C-SENSE6.5 acquisition provided almost the same image quality, and C-SENSE8 resulted in subtly decreased image quality for vessel visualization compared with that of SENSE4.5. However, the source image quality in C-SENSE5, 6.5, and 8 acquisitions was better than that obtained with SENSE4.5 acquisition. In dynamic flow analysis, the ATT values of all C-SENSE factors had a strong correlation with SENSE4.5. Moreover, the acquisition times of C-SENSE6.5 and C-SENSE8 were reduced by 18 and 37%, respectively, compared with those obtained using SENSE4.5 and C-SENSE5.

Compressed sensing has been used to accelerate vessel-selective ASL-based angiography without loss of image quality based on 2D golden-angle radial acquisition (11), which is a non-Cartesian sampling method and is not widely available for clinical use. Obara et al. demonstrated that 4D-PACK acceleration using SENSE and pCASL combined with Keyhole and View-sharing shows inflow dynamics similar to those of 4D-pCASL and provides better peripheral visualization than CINEMA-based 4D MRA (14). However, the maximum reduction factor of SENSE is limited by the number of independent coil elements or coil geometry. Image artifacts may appear as ghosts inside or outside the ROI if the SENSE acceleration factor is too high (23). A previous study mentioned that specked noise and SENSE artifacts were observed in the central area of 3D time-of-flight (TOF) MRA images with a large SENSE factor = 4, while these artifacts were rarely found on the images acquired by C-SENSE acceleration (24). In this study, the source images of 4D-PACK with C-SENSE with 5, 6.5, and 8 showed reduced artifacts compared with the artifacts present when SENSE4.5 was used. Additionally, the results demonstrated that 4D-PACK with C-SENSE could avoid artifacts in reconstructed images using SENSE.

Vessel visualization of the M1 to M3 segments was not significantly different in all sequences. The CNRs of M1 to M3 segments were not significantly different in C-SENSE5 and 6.5 acquisition compared with SENSE4.5, but higher than C-SENSE8 and C-SENSE10. The CNRs in the M1, M2, and M3 segments were relatively high in all acceleration factors, especially for SENSE4.5, C-SENSE5, 6.5, and 8; these high CNRs may have limited influence on clinical diagnosis. Evaluation at the M4 segment of the MCA is important for distal artery evaluation. Togao O et al. reported that 4D-PACK is sensitive in identifying distal arteries with slow flow (5). The CNRs and vessel visualization of the M4 segment were not significantly different in the C-SENSE5 and 6.5 acquisitions compared with SENSE4.5, which may indicate that C-SENSE6.5 acquisition could be used to evaluate the distal arteries at the M4 segment. However, the margins of the terminal vessels were gradually blurred, and the CNRs were gradually reduced as the acceleration factor increased to C-SENSE8 and 10. Therefore, C-SENSE8 could be used for the clinical diagnosis of ailments of the M4 segment, although further clinical studies should be performed for verification.

The 4D-PACK with different C-SENSE factors had high ATT reproducibility with SENSE4.5 in MCA, which indicated that C-SENSE acquisition had reliable hemodynamic information. In patients with cerebrovascular disease, ATT will increase because of slow blood flow. Whether there is a relationship between increased ATT and cerebrovascular events could be explored in the future.

The 4D-PACK acquisition with all acceleration factors had a similar diagnostic value for cerebral AVM in this study compared with DSA images, although the vessel signal determined by C-SENSE10 was slightly lower. The feeding artery, nidus, and draining vein could be shown in 4D-PACK images individually. In addition, all sequences had a similar nidus size. Previous studies also reported that 4D-PACK had high sensitivity and specificity for detecting venous drainage and the feeding artery in patients with cerebral AVM (15). Pertinently, vessel visualization of the distal arteries is important in patients with MMD. Moreover, NCE 4D MRA is useful in vascular architecture evaluation of the leptomeningeal vessels in MMD (5). In this study, most of the distal collaterals in MMD patients appeared in all 4D-PACK images compared with DSA images. C-SENSE5 and C-SENSE6.5 acquisition provided similar vessel visualization of the distal arteries as SENSE4.5, C-SENSE8 revealed vague margins of the distal arteries, and C-SENSE10 showed only a few distal arteries.

There were several limitations in this study. First, patients with different vasculopathies, which may influence the dynamic data, were not included. A patient with AVM and five patients with MMD were assessed in this study. Notably, it seems that 4D-PACK combined with all acceleration factors can be used to diagnose issues with feeding arteries, niduses, and draining veins in patients with cerebral AVM. Additionally, SENSE4.5, C-SENSE5, and C-SENSE6.5 acquisitions can be used to diagnose problems in the distal arteries in patients with MMD. However, this possibility should be confirmed by more clinical studies. Second, the sample size was relatively small. However, the diagnostic power of one-way ANOVA of the CNR in the M1 segment was above 0.9, as assessed with the G^*^Power 3.1.9.4 software. Third, the assessed ROIs were located in four segments of the unilateral MCA, while the remaining intracranial and extracranial arteries were not analyzed. MCA is one of the major intracranial arteries and includes fast and slow flow circulation, which could be a reflection of intracranial hemodynamic characteristics and image quality.

## Conclusion

In conclusion, C-SENSE8 acquisition can be used for routine screening and clinical diagnosis of intracranial macrovascular disease (atherosclerotic stenosis, aneurysm, etc.), which could reduce the acquisition time by 37% (3 min 54 s) with balanced image quality when NCE 4D MRA is used compared with SENSE4.5 acceleration. C-SENSE6.5 acquisition could be used to evaluate both the intracranial macrovascular and distal arteries, which could reduce the acquisition time by 18% (5 min 5 s). This excellent acceleration advantage of C-SENSE could improve the clinical application of 4D MRA.

## Data Availability Statement

The original contributions presented in the study are included in the article/supplementary material, further inquiries can be directed to the corresponding author.

## Ethics Statement

The studies involving human participants were reviewed and approved by Medical Ethics Committee of the Nanjing Drum Tower Hospital. The patients/participants provided their written informed consent to participate in this study.

## Author Contributions

MW, YM, and BZ designed the study. MW performed the statistical analyses. MW and YM contributed to data preparation and drafting the original manuscript. JZ and YM were responsible for MR scanning. FZ and FC evaluated the MR images. FC managed the subject recruitment. BZ modified and confirmed the final article. All authors contributed to the article and approved the submitted version.

## Funding

This work was supported by the National Natural Science Foundation of China (81720108022, BZ, 81971596, Xin Zhang, 81701672, Zhao Qing); the Social Development Project of Science and Technology Project in Jiangsu Province (BE2017707); Key Medical Talents of the Jiangsu province, the 13th Five-Year Health Promotion Project of the Jiangsu province (ZDRCA2016064); the Project of the Sixth Peak of Talented People (WSN−138). The funders had no role in the study design, data collection and analysis, decision to publish, or preparation of the manuscript.

## Conflict of Interest

JZ was employed by Philips Healthcare, Shanghai, China. The remaining authors declare that the research was conducted in the absence of any commercial or financial relationships that could be construed as a potential conflict of interest.

## Publisher's Note

All claims expressed in this article are solely those of the authors and do not necessarily represent those of their affiliated organizations, or those of the publisher, the editors and the reviewers. Any product that may be evaluated in this article, or claim that may be made by its manufacturer, is not guaranteed or endorsed by the publisher.
